# Micro-Doppler Ambiguity Resolution for Wideband Terahertz Radar Using Intra-Pulse Interference

**DOI:** 10.3390/s17050993

**Published:** 2017-04-29

**Authors:** Qi Yang, Yuliang Qin, Bin Deng, Hongqiang Wang, Peng You

**Affiliations:** College of Electronic Science and Engineering, National University of Defense Technology, Changsha 410073, China; yangqi_nudt@163.com (Q.Y.); dengbin@nudt.edu.cn (B.D.); oliverwhq@tom.com (H.W.); ypnudt@126.com (P.Y.)

**Keywords:** terahertz radar, micro-Doppler ambiguity resolution, intra-pulse interference, micro-motion parameter estimation

## Abstract

Micro-Doppler, induced by micro-motion of targets, is an important characteristic of target recognition once extracted via parameter estimation methods. However, micro-Doppler is usually too significant to result in ambiguity in the terahertz band because of its relatively high carrier frequency. Thus, a micro-Doppler ambiguity resolution method for wideband terahertz radar using intra-pulse interference is proposed in this paper. The micro-Doppler can be reduced several dozen times its true value to avoid ambiguity through intra-pulse interference processing. The effectiveness of this method is proved by experiments based on a 0.22 THz wideband radar system, and its high estimation precision and excellent noise immunity are verified by Monte Carlo simulation.

## 1. Introduction

Terahertz (THz) waves usually refer to electromagnetic waves with frequencies varying from 0.1 to 10 THz. The terahertz band lies between the millimeter wave and infrared, and it is a transitional band from electronics to photonics. Its position in the spectrum confers special properties and applications on terahertz waves that differ from other bands [1,2,3]. In recent years, the terahertz radar technology has developed rapidly with breakthroughs in terahertz sources, signal detectors, and other devices, and many terahertz radar systems have been established, mainly for research on high-resolution imaging [4,5]. However, research on micro-motion targets, as a common type of object in the real world, is still very limited. Although terahertz waves, due to their short wavelengths, are more sensitive to micro-Doppler than microwaves and hence more suited for micro-Doppler resolution. Despite the advantages mentioned, micro-Doppler resolution in the terahertz band faces a significant hurdle, i.e., the micro-Doppler ambiguity induced by the inadequate pulse repetition frequency (PRF) of the radar system. The limited PRF sets an upper limit to the maximal observable micro-Doppler values. Specifically, the micro-Doppler can be unambiguously observed only when it lies in the interval of –PRF/2 to PRF/2, and it will be ambiguous outside this interval. In addition, micro-Doppler in the terahertz band inclines to be ambiguous due to the carrier frequency that is higher than the microwave band.

For micro-motion in the terahertz band, Jin Li et al. performed a theoretical analysis of typical micro-motion forms to establish the micro-Doppler signature model, and then compared the micro-Doppler effect in the terahertz band and that in the X band by using the joint time–frequency analysis method [6]. Zhengwu Xu et al. analyzed the characteristics of micro-motion and investigated methods for motion parameter estimation and micro-Doppler signature extraction by the Radon transform [7]. However, micro-Doppler ambiguity resolution was not considered, just because the micro-Doppler values they assumed are relatively smaller than PRF/2. In practice, micro-Doppler ambiguity in the terahertz radar system is inevitable, so these algorithms are inapplicable for our concerns. For micro-Doppler ambiguity resolution, Zhuang et al. proposed a method based on short-time compressed sensing to solve micro-Doppler ambiguity [8]. The method is based on a sparse probing pulse train with its transmitting time random, which requires high demands on the hardware and is hard to achieve in the terahertz radar system. In other words, the method based on short-time compressed sensing is inapplicable for our concerns of a fixed PRF situation.

Therefore, we propose a micro-Doppler ambiguity resolution method for wideband terahertz radar using intra-pulse interference in this paper. Its essence lies in converting the carrier frequency to a relatively small value by frequency interference between the sampling points in a pulse or a frequency-sweep period to avoid ambiguity. Compared with the existing Doppler ambiguity resolution methods, such as using the multiple PRF radar or the multi-channel radar [8,9,10], the method proposed in this paper enjoys easy realization and little computational work, and does not increase the complexity of the system. The paper is organized as follows: the theoretical signal model of micro-motion targets is established in Section 2, and the intra-pulse interference processing is introduced in detail. In Section 3, a 0.22 THz experimental radar system is introduced, and experiments based on two four-sided corner reflectors are carried out. In Section 4, the micro-Doppler ambiguity resolution method using intra-pulse interference is applied to experimental data of the terahertz radar system, and its excellent performance is verified by theory analysis and Monte Carlo simulation. Conclusions are presented in the last section.

## 2. Theory Analysis

### 2.1. Radar Echo Signal Model of Micro-Motion Targets

Due to the intra-pulse interference processing in this method, a wideband signal based on a linear frequency modulated (LFM) pulse mode or a frequency-modulated continuous wave (FMCW) mode is needed, and the transmitting signal can be expressed as follows:

(1) $$s(\hat{t},t_{m})=rect\left(\frac{\hat{t}}{T_{p}}\right)\exp\left[j2\pi \left(f_{c}t+\frac{1}{2}\gamma {\hat{t}}^{2}\right)\right]$$

where $\hat{t}$ and $t_{m}$ represent the range fast-time and azimuth slow-time, respectively. $T_{p}$ is the pulse width in the LFM mode or the frequency-sweep period in the FMCW mode. $f_{c}$ is the carrier frequency of the radar system, and γ is the chirp rate.

Typical micro-motions of radar targets include precession, rotation, vibration, etc. Their projections on the radar slight can be considered as simple harmonic motions if translation between radar and mass center of targets is excluded [11]. The motion model of a micro-motion target with K scattering centers can be expressed as

(2) $$R_{k}=R_{0}+a_{k}\sin(\omega_{k}t_{m}+\varphi_{k}),k=1,2...K$$

where $a_{k}$ and $\omega_{k}$ are the amplitude and angular velocity of the micro-motion scattering center k, respectively. $\varphi_{k}$ is the initial phase that characterizes the relationship between radar slight and the initial position of the scattering center. $R_{0}$ denotes the initial distance between the radar and the target. Thus, the echo signal of the micro-motion target can be expressed as follows:

(3) $$s_{r}(\hat{t},t_{m})=\sum_{k=1}^{K}rect\left(\frac{t-2R_{k}/c}{T_{p}}\right)\exp\left[j2\pi \left(f_{c}(t-\frac{2R_{k}}{c})+\frac{1}{2}\gamma {\left(\hat{t}-\frac{2R_{k}}{c}\right)}^{2}\right)\right]$$

where c is the speed of light. According to the definition of Doppler, the micro-Doppler of each micro-motion scattering center is

(4) $$f_{dk}=\frac{2\omega_{k}f_{c}a_{k}}{c}\cos(\omega_{k}t_{m}+\varphi_{k}),k=1,2...K.$$

Therefore, it is clear that the micro-Doppler of each scattering center on the micro-motion target is sinusoidal modulated, and its maximum value depends on the carrier frequency, the amplitude, and the angular velocity of micro-motion. In effect, the micro-Doppler is in proportion to the carrier frequency. The higher the carrier frequency, the more evident the micro-Doppler effect becomes. Therefore, micro-Doppler values in the terahertz band are far larger than that in the microwave band under identical micro-motion conditions. Suppose the PRF of the transmitting signal is 1000 Hz, and the maximum micro-Doppler value of a micro-motion scattering center is about 100 Hz when the carrier frequency is 10 GHz. At 0.22 THz, it would reach 2200 Hz, which substantially exceeds the upper limit of PRF/2; hence, ambiguity is present. In addition, the wideband radar system itself has a certain range resolution owing to its bandwidth, especially for the terahertz radar system. However, for two micro-motion targets located at a difference range of ΔR, their micro-Doppler difference will be $2\omega f_{c}\Delta R/c$ from Equation (4), which will be far greater than ΔR in the terahertz band. In other words, the micro-Doppler resolution is much more sensitive than the range resolution, and that is the main advantage of the micro-Doppler resolution as long as the ambiguity problem is solved, which is exactly the focus of this paper.

In the signal receiving and data acquisition respects, the transmitting and receiving signals possess a large product of time-width and bandwidth, which require a high performance of hardware if sampling and processing directly according to the Nyquist sampling theorem. Consequently, the dechirp method, i.e., mixing the echo signal with a reference signal, which usually refers to the echo signal of a target located at the reference distance $R_{ref}$, is often adopted, and it can be expressed as follows:

(5) $$s_{ref}(\hat{t},t_{m})=rect\left(\frac{t-2R_{ref}/c}{T_{p}}\right)\exp\left[j2\pi \left(f_{c}(t-\frac{2R_{ref}}{c})+\frac{1}{2}\gamma {\left(\hat{t}-\frac{2R_{ref}}{c}\right)}^{2}\right)\right].$$

The intermediate frequency signal after dechirp is

(6) $$\begin{array}{l} s_{if}(\hat{t},t_{m})=s_{r}(\hat{t},t_{m})\cdot s^{*}{}_{ref}(\hat{t},t_{m})\\ =\sum_{k=1}^{K}rect\left(\frac{\hat{t}-2R_{k}/c}{T_{p}}\right)\exp\left[-j\frac{4\pi }{c}\gamma \left(\hat{t}-\frac{2R_{ref}}{c}\right)R_{\Delta k}-j\frac{4\pi }{c}f_{c}R_{\Delta k}+j\frac{4\pi }{c^{2}}R_{\Delta k}^{2}\right] \end{array}$$

where $R_{\Delta }=R_{k}-R_{ref}$, and $R_{ref}$ is usually set to $R_{0}$. The last two phase terms in Equation (6) are the residual video phase (RVP) term and the range skew term respectively, and they are easily compensated because the range profiles are Sinc functions with very narrow widths. After phase compensation, the Equation (6) can be rewritten as follows:

(7) $$s_{if}(\hat{t},t_{m})=\sum_{k=1}^{K}rect\left(\frac{\hat{t}-2R_{k}/c}{T_{p}}\right)\exp\left[-j\frac{4\pi }{c}\gamma \left(\hat{t}-\frac{2R_{ref}}{c}\right)a_{k}\sin(\omega_{k}t_{m}+\varphi_{k})\right].$$

### 2.2. Micro-Doppler Ambiguity Resolution Based on Intra-Pulse Interference

If we rewrite the signal of Equation (7) in matrix form, the columns of the matrix represent pulses varying with time, and the rows of the matrix represent sampling points of the pulses or the frequency-sweep periods. In this way, each row of the signal matrix can be treated as the echo signal based on a signal frequency system, and its expression is as follows:

(8) $$s_{i}(t)=\sum_{k=1}^{K}\exp\left[-j\frac{4\pi \gamma {\hat{t}}_{i}a_{k}}{c}\sin(\omega_{k}t+\varphi_{k})\right],i=1,2...N,k=1,2...K$$

where N is the number of sampling points of the pulses or the frequency-sweep periods, and ${\hat{t}}_{i}$ is the sampling time of the i-th sampling point. The equivalent carrier frequency of the i-th row of the signal matrix is $f_{i}=\gamma {\hat{t}}_{i}$, where $f_{i}$ locates in the interval of $f_{c}-B/2$ to $f_{c}+B/2$.

In order to achieve micro-Doppler ambiguity resolution, a signal vector is obtained by conjugate multiplying the i-th row and the j-th row of the signal matrix, as shown below.

(9) $$\begin{array}{l} s_{i,j}(t)=s_{i}(t)\cdot s^{*}{}_{j}(t)=\sum_{k=1}^{K}\exp\left[-j\frac{4\pi \gamma \left({\hat{t}}_{i}-{\hat{t}}_{j}\right)a_{k}}{c}\sin(\omega_{k}t+\varphi_{k})\right]\\ \;+\sum_{\begin{array}{l} m=1,2...K\\ n=1,2...K\\ m\neq n \end{array}}\exp\left[-j\frac{4\pi \gamma }{c}\left(a_{m}{\hat{t}}_{i}\sin(\omega_{m}t+\varphi_{m})-a_{n}{\hat{t}}_{j}\sin(\omega_{n}t+\varphi_{n})\right)\right] \end{array}.$$

It is obvious from Equation (9) that the Doppler information of the micro-motion scattering centers are included in the first K terms, and the remaining terms can be treated as cross terms that need to be eliminated as far as possible. To suppress the cross term in Equation (9), herein we set i=1,2...N/2 and j=N/2+1,N/2+2...N. Then, we conjugate multiply the i-th and the j-th rows of the signal matrix to obtain N/2 signal vectors, and coherent accumulation is carried out between the signal vectors to obtain a new signal, s(t):

(10) $$s(t)\approx \sum_{i=1}^{N/2}\sum_{k=1}^{K}\exp\left[-j\frac{4\pi \gamma \left({\hat{t}}_{i}-{\hat{t}}_{i+N/2}\right)a_{k}}{c}\sin(\omega_{k}t+\varphi_{k})\right]=\frac{N}{2}\sum_{k=1}^{K}\exp\left[-j\frac{2\pi Ba_{k}}{c}\sin(\omega_{k}t+\varphi_{k})\right].$$

At this point, the micro-Doppler distribution of each micro-motion scattering center can be obtained by time–frequency analysis of the signal in Equation (11), and its theoretical expression through intra-pulse interference is as follows:

(11) $$f_{dk}^{\prime }=\frac{\omega_{k}Ba_{k}}{c}\cos(\omega_{k}t_{m}+\varphi_{k}),k=1,2...K.$$

**Figure 1:**
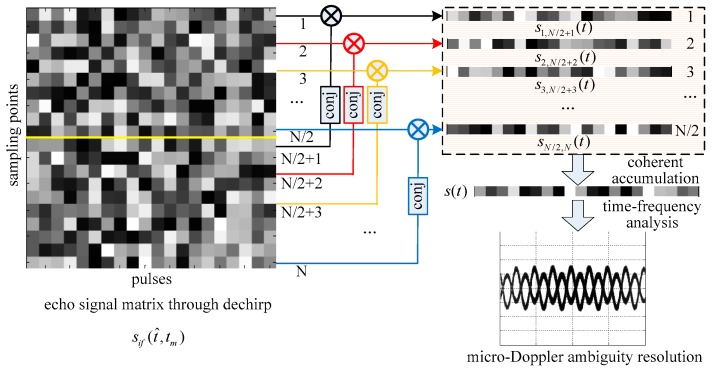
Schematic diagram of the micro-Doppler ambiguity resolution method.

Compared with the micro-Doppler expression in Equation (4), the intra-pulse interference processing converts $f_{c}$ in the micro-Doppler expression into an equivalent value B/2. In other words, the micro-Doppler value has been shrunk $2f_{c}/B$ times to avoid ambiguity through intra-pulse interference processing. Nevertheless, the method proposed in this paper does not completely solve the problem of micro-Doppler ambiguity, and what it does is extend the upper limit of ambiguity from PRF/2 to its $2f_{c}/B$ times, which is large enough to avoid micro-Doppler ambiguity in many applications. The schematic diagram of the micro-Doppler ambiguity resolution method for wideband terahertz radar using intra-pulse interference is shown in Figure 1.

After micro-Doppler ambiguity resolution is implemented, parameter estimation of the micro-motion target will become relatively simple. The traditional methods based on circular correlation coefficients or sparse recovery for period estimation [12,13], as well as inverse Radon transform [8] and Fourier–Bessel transform [14] for micro-Doppler and initial phase estimation, will also be available.

## 3. The Wideband Terahertz Radar System and Experiments

### 3.1. The Terahertz Radar System

A 0.22 THz radar system was established, and experiments were carried out to verify the micro-Doppler ambiguity resolution method proposed in this paper. The terahertz radar system mainly consists of four modules: the signal source, the RF chains, intermediate frequency (IF) module, and the data collection module. The signal source module consists of a direct digital waveform synthesis (DDWS), a phase locked loop (PLL) and two coherent local oscillators with a difference frequency of 60 MHz. The PLL can generate signals that have characteristics of broadband, low spur, and low phase noise through rapidly changing the frequency dividing factor [15]. An initial signal range from 2.45 to 3.25 GHz is generated by the DDWS and the PLL, and is then modulated onto the coherent local oscillators to output the IF signal varying from 13.45 to 14.25 GHz. The mode of the IF signal can be continuous wave (CW), FMCW, or others. For the sake of intra-pulse processing in this method, we choose the FMCW mode, and its average output power is about 100 mW.

**Figure 2:**
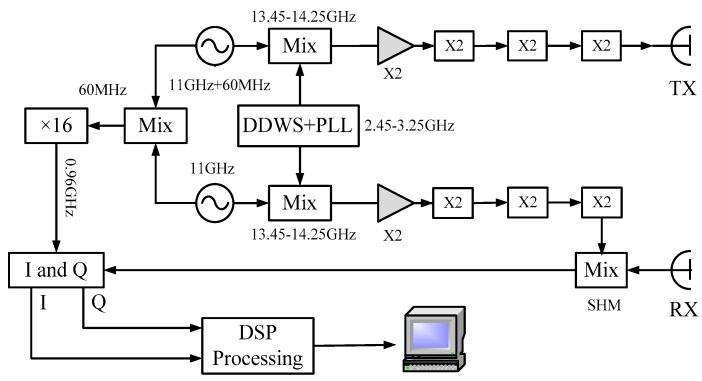
Schematic diagram of the 0.22 THz radar system.

**Figure 3:**
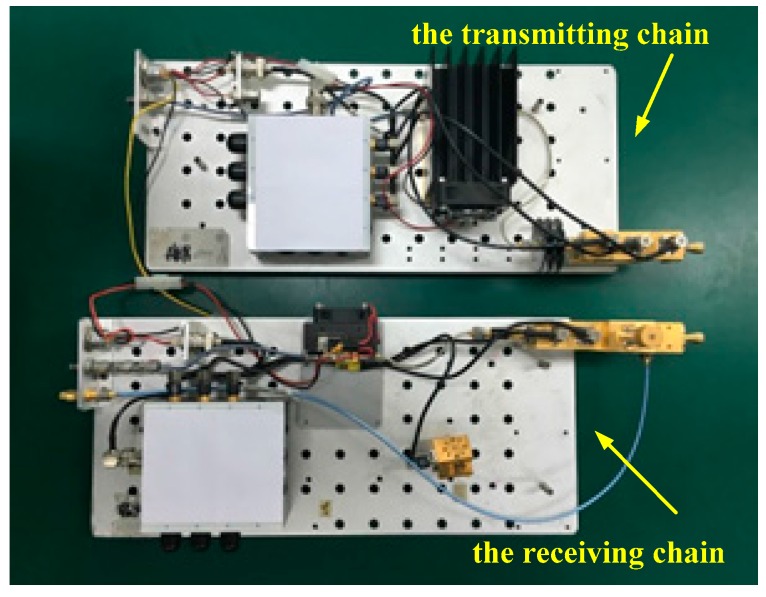
The transmitting and receiving front-ends of the 0.22 THz radar system.

The RF chains are the key design of the terahertz radar system. They consist of the transmitting chain and the receiving chain. In the transmitting chain, the Ku-band IF signal is multiplied into the terahertz band after power amplifying and frequency multiplications, and the terahertz signal is then transmitted by a conical horn antenna. Because we set the frequency range of the IF signal to 13.45 to 14.25 GHz, the carrier frequency of this radar system is about 0.22 THz, and the bandwidth is 12.8 GHz. Through harmonic mixing in the receiving chain, the received terahertz signal is down-converted to IF for super heterodyne reception. In order to obtain high sensitivity and a large dynamic range of the system, a high precision controllable digital attenuator with a maximum attenuation of 30 dB is added to the receiving chain. Through the sub-harmonic mixer (SHM) in the receiving chain, the terahertz signal is down-converted to baseband and demodulated by an I/Q demodulator for A/D sampling. The data acquisition and control electronics are based upon a signal processing board with a 14 bit A/D-card with a sampling rate of 40 MHz/s. Finally, Ethernet transmits the collected data to the PC for further processing. Both transmitting and receiving signals are controlled by a PC via the PC serial ports and the control software. The schematic diagram of the 0.22 THz radar system is shown in Figure 2, and its transmitting and receiving front-ends are shown in Figure 3.

### 3.2. The Experiments on Rotating Targets

**Figure 4:**
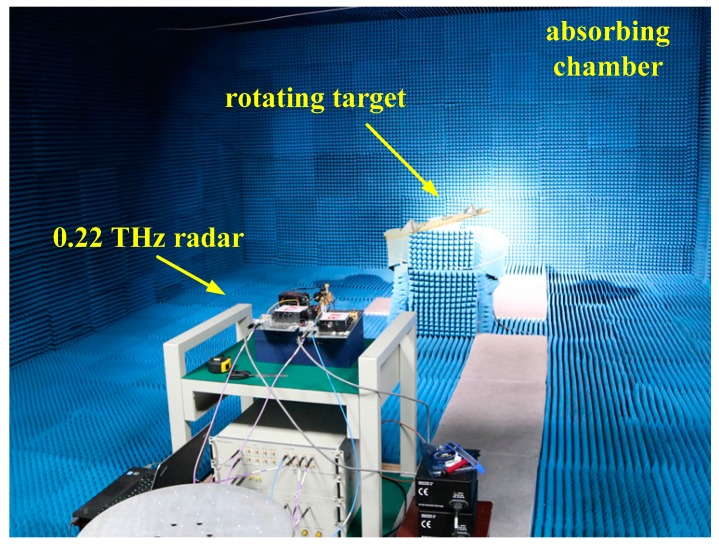
The experimental scenario.

**Figure 5:**
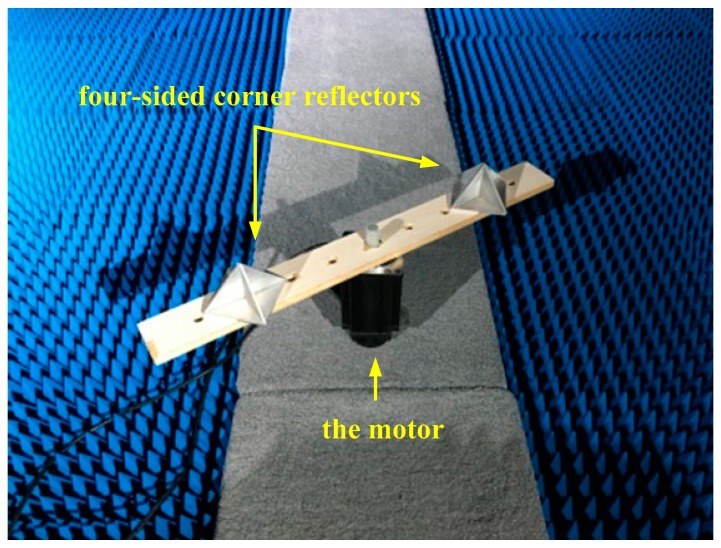
The rotating targets.

The targets are two rotating four-sided corner reflectors driven by a motor, and the four-sided corner reflectors can be seen as ideal point targets. The rotation angular velocity of the motor is precisely adjustable from 0 to 500 r/min, and the rotation amplitudes are 16 and 24 cm, respectively. The distance between the terahertz radar system and the rotating target is set to 5 m. The motor is covered with absorbing material, and all other devices are placed in an absorbing chamber to minimize the noise of the background. In the experiments, the sampling point of each frequency-sweep period is set to 4096, and the PRF of the signal is set to 1000 Hz. The experimental scenario and the rotating targets are shown in Figure 4 and Figure 5.

## 4. Experimental Results and Analysis

### 4.1. The Experiment Results

Three experiments on the rotating corners reflectors are carried out to verify the micro-Doppler ambiguity resolution method proposed in this paper, and the angular velocities of the targets in the three experiments are 20 r/min, 40 r/min, and 60 r/min, respectively. It should be noted that this method is capable of handling targets that move at more than one frequency because the essence of this method is reducing the micro-Doppler values $2f_{c}/B$ times to avoid ambiguity through intra-pulse interference processing, and it has nothing to do with the frequencies of targets. Limited by the current devices and conditions of our laboratory, the experimental targets move at one frequency, but the method can be fully applicable to the multi-frequency situations. In the experiments, the sampling point of each frequency-sweep period is set to 4096, and the PRF of the signal is set to 1000 Hz. In such a situation, Sampling Point 1 is interfered with by Sampling Point 2049, and Sampling Point 2 is interfered with by Sampling Point 2050, and so on, for each of the 2048 pairs of sampling points.

**Figure 6:**
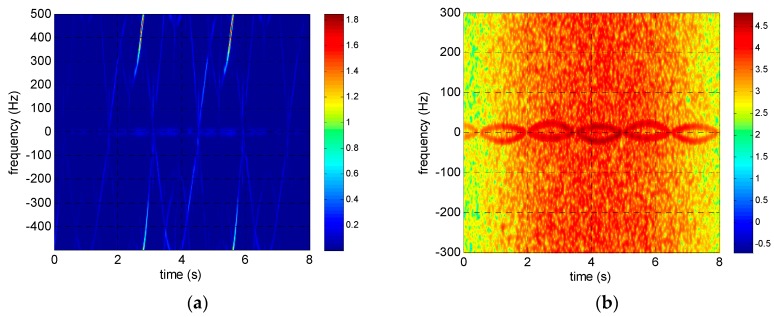
Time–frequency distributions of corner reflectors rotating at 20 r/min (**a**) before and (**b**) after intra-pulse interference processing.

**Figure 7:**
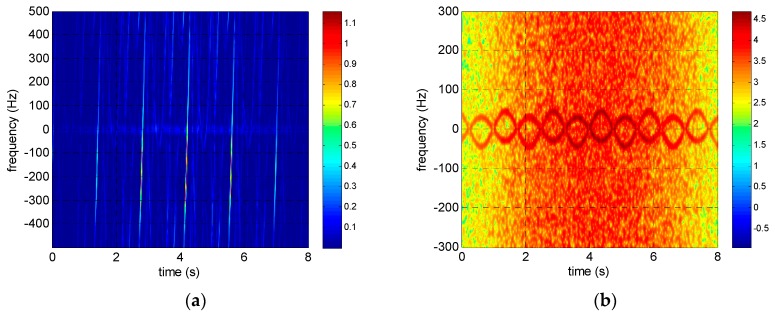
Time–frequency distributions of corner reflectors rotating at 40 r/min (**a**) before and (**b**) after intra-pulse interference processing.

**Figure 8:**
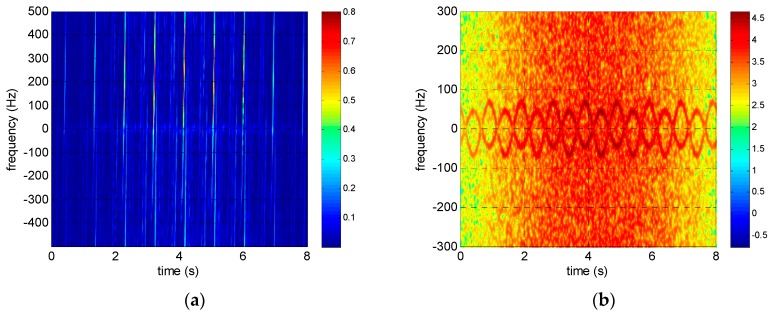
Time–frequency distributions of corner reflectors rotating at 60 r/min (**a**) before and (**b**) after intra-pulse interference processing.

The reassigned smoothed pseudo Wigner–Ville distribution (RSPWVD) is adopted in both simulations and experiments in this paper for time–frequency analysis because it has both good time–frequency concentration and low cross-term disturbance. The time–frequency distributions of the rotating targets before and after intra-pulse interference processing are shown in Figure 6, Figure 7 and Figure 8. It is obvious that the time–frequency distributions are seriously ambiguous in the terahertz band because of its excessive micro-Doppler value. However, the micro-Doppler is shrunk $2f_{c}/B$ times to avoid ambiguity through intra-pulse interference processing, and the time–frequency curves modulated by sinusoidal functions are clearly visible.

After micro-Doppler ambiguity resolution is implemented, we adopted the autocorrelation method in time domain for micro-motion period estimation, and inverse Radon transform for micro-Doppler and initial phase estimation in the following simulation and experiments owing to their high accuracy and robustness. The theoretical micro-Doppler values, the estimated micro-Doppler values and their relative errors of the corner reflector rotating at 24 cm are shown in Table 1.

### 4.2. Performance Analysis

**Figure 9:**
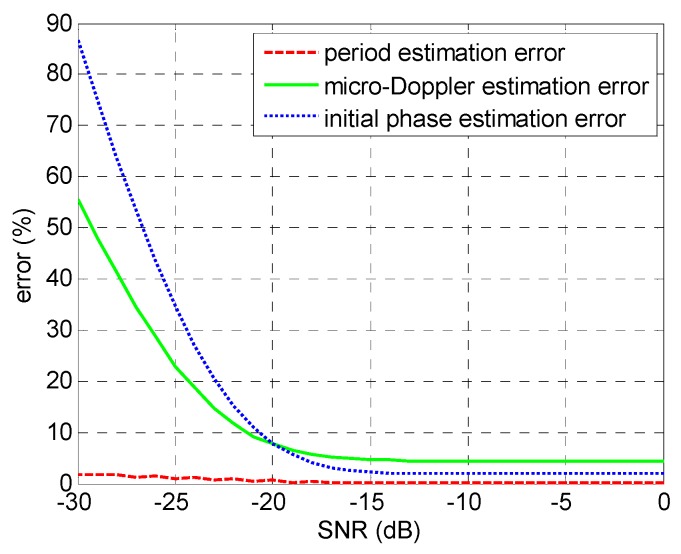
Relationships between signal-to-noise ratio (SNR) and the parameter estimation errors.

The signal-to-noise ratio (SNR) of the echo signal is generally an important factor for parameter estimation accuracy; however, it has a limited impact on the method proposed in this paper, primarily because of the good noise immunity performance of several operations such as the autocorrelation, the coherent accumulation, and the inverse Radon transform. By Monte Carlo simulation, the relationships between SNR of the echo signal and the parameter estimation errors are qualitatively shown in Figure 9. The target in simulation is two scattering centers rotating at 16 cm and 24 cm, respectively, just like the experimental scenario in Section 3. The SNR of the echo signal ranges from −30 to 0 dB. As can be seen from Figure 9, the method proposed in this paper is of good anti-noise ability, especially when the SNR is greater than −17 dB. As periodically of the signal or its time–frequency is less affected by the signal noise, micro-motion period estimation error keeps a low level from −30 to 0 dB. The micro-Doppler estimation error and the initial phase estimation error are maintained within 5% when the SNR is greater than −17 dB. Below −17 dB, each signal is seriously contaminated by noise, and the time–frequency analysis and inverse Radon transform cannot work effectively, despite the adoption of intra-pulse interference processing.

## 5. Conclusions

Research on parameter estimation of micro-motion targets in the terahertz band are of great value to exploit the advantages of the terahertz band and promoting the applications of terahertz radars, while micro-Doppler ambiguity is an inevitable problem. In this paper, we analyzed the reason of micro-Doppler and proposed a micro-Doppler ambiguity resolution method for wideband terahertz radar using intra-pulse interference. Its essence is shrinking the micro-Doppler values several dozen times to avoid ambiguity through intra-pulse interference processing, in other words, extending the upper limit of ambiguity several dozen times. In addition, a 0.22 THz radar system was adopted, and experiments on rotating corner reflectors were carried out. The experimental results and Monte Carlo simulation demonstrated the high estimation precision and excellent noise immunity of this method, which is determined by the intra-pulse interference and coherent accumulation processing. Especially when the SNR is above −17 dB, micro-motion parameter estimation errors can be controlled within 5%.

## Figures and Tables

**Table 1:** Comparison between theoretical values and estimated values of micro-Doppler value of the corner reflector rotating at 24 cm.

	20 r/min	40 r/min	60 r/min
theoretical micro-Doppler values (Hz)	742.59	1485.18	2227.77
micro-Doppler values after intra-pulse interference (Hz)	21.45	42.89	64.34
estimated micro-Doppler values (Hz)	22.10	44.47	66.58
relative errors (%)	3.03	3.68	3.48
